# Billroth II with Braun Enteroenterostomy Is a Good Alternative Reconstruction to Roux-en-Y Gastrojejunostomy in Laparoscopic Distal Gastrectomy

**DOI:** 10.1155/2017/1803851

**Published:** 2017-01-09

**Authors:** Long-Hai Cui, Sang-Yong Son, Ho-Jung Shin, Cheulsu Byun, Hoon Hur, Sang-Uk Han, Yong Kwan Cho

**Affiliations:** Department of Surgery, Ajou University School of Medicine, Suwon, Republic of Korea

## Abstract

*Background*. Although Billroth II (BII) reconstruction is simpler and faster than Billroth I or Roux-en-Y (RY) reconstruction in patients undergoing totally laparoscopic distal gastrectomy (TLDG), BII reconstruction is associated with several complications, including more severe bile reflux. BII Braun anastomosis may be a better alternative to RY reconstruction.* Methods*. This retrospective study included 56 consecutive patients who underwent TLDG for gastric cancer, followed by BII Braun or RY reconstruction, between January 2013 and December 2015. Surgical outcomes, including length of operation, quantity of blood lost, and postoperative complications, were compared in the two groups.* Results*. Clinicopathological characteristics did not differ between the BII Braun and RY groups. Mean length of operation was significantly longer in the RY than the BII Braun group (157.3 min versus 134.6 min,* p* < 0.010), but length of hospital stay, blood loss, and complication rate did not differ between the two groups. Ileus occurred in three patients (10.0%) in the RY group. Endoscopic findings 6 months after surgery showed bile reflux in seven (28%) patients in the BII Braun group and five (17.2%) in the RY group (*p* = 0.343), but no significant differences in rate of gastric residue or degree of gastritis in the remnant stomach in the two groups.* Conclusions*. B-II Braun anastomosis is a good alternative to RY reconstruction, reducing length of operation and ileus after TLDG.

## 1. Introduction

Gastric cancer is one of the most common cancers and the third leading cause of cancer-related deaths worldwide [1]. Surgical resection remains the only definitive treatment of this malignant disease [2]. Early diagnosis of gastric cancer has resulted in a significant improvement in the long-term survival of patients undergoing surgery [3].

Some patients who undergo surgery for gastric cancer experience postgastrectomy complications, including malabsorption, dumping syndrome, reflux esophagitis, alkaline gastritis, and delayed gastric emptying [4–6]. Reflux gastritis, which occurs mainly after Billroth II (BII) reconstruction, causes long-term distress, impairs patient quality of life, and may lead to increased risk of metachronous cancer development [7, 8]. The introduction of Roux-en-Y (RY) reconstruction dramatically reduced the rate of alkaline reflux gastritis [9–13]. Early series reported nearly universal success after Roux diversion, resulting in the suggestion that RY reconstruction be considered a method of primary reconstruction after gastrectomy [14]. However, RY reconstruction has drawbacks, including difficulties performing the procedure and severe complications, such as Roux limb stasis and internal herniation [15–18].

The development of laparoscopic techniques increased the number of patients undergoing totally laparoscopic distal gastrectomy (TLDG) with intracorporeal anastomosis. Use of this surgical method increased the percentage of patients undergoing BII reconstruction, as it is both simple and rapid. BII reconstruction still has limitations, as it can cause more severe bile reflux, increasing the risk of metachronous cancer development [19, 20]. Braun [21] introduced an enteroenterostomy anastomosis in an attempt to divert food from the afferent limb, thus reducing the incidence of the “vicious circle” syndrome. This simple and easy method may be used as a standard method, at least for older patients undergoing TLDG.

It is unclear whether BII Braun anastomosis results in superior perioperative outcomes when compared with RY reconstruction. Few studies to date have compared short-term outcomes and endoscopic findings after 6 months in patients undergoing laparoscopic intracorporeal anastomosis using these two methods. This study therefore compared the short-term surgical outcomes of BII Braun anastomosis and RY reconstruction in patients who underwent laparoscopic distal gastrectomy (LDG) performed by a single surgeon.

## 2. Material and Methods

### 2.1. Study Design and Patients

A total of 376 consecutive patients at Ajou University Hospital underwent TLDG by a single surgeon between January 2013 and December 2015. Of these, 167 patients who underwent Billroth I (BI) reconstruction were excluded, as were 153 patients who underwent BII anastomosis alone without the Braun procedure. Of the remaining 56 patients, 26 underwent BII Braun reconstruction and 30 underwent RY reconstruction. Findings in these two groups were evaluated retrospectively.

The evaluated parameters included patient demographics, comorbidities, operative details, time to first flatus, time to sips of water, length of hospital stay, and postoperative complications. Tumor depth, nodal status, and stage were classified according to the 7th American Joint Committee on Cancer Staging System. Lymph node dissection was performed according to the Guidelines of the Japanese Gastric Cancer Association. All patients underwent follow-up upper gastrointestinal endoscopy 6 months postoperatively. Gastric residue, degree of gastritis, and bile reflux (RGB) classification was analyzed, with higher scores indicating worse symptoms or signs in the remnant stomach [22].

The study was reviewed and approved by the Ajou University Hospital Institutional Review Board.

### 2.2. Surgical Technique

LDG was performed with the patient in the supine position under general anesthesia. The operator and endoscopist stood on the right side of the patient and the first assistant stood on the left side. The method used one 10 mm port for the laparoscope, as well as two 12 mm and two 5 mm ports. The pneumoperitoneum was maintained between 10 and 13 mmHg. Ultrasonically activated shears were used for lymph node dissection.

All patients in our center have undergone reconstruction using intracorporeal anastomosis since April 2010. Following LDG, patients in the BII Braun group underwent gastrojejunostomy about 40 cm from the ligament of Treitz in antecolic and isoperistaltic manners. Braun anastomosis was performed about 25 cm distal to the gastrojejunostomy, using a linear stapler 60 mm in length with white cartilage. Then, entry hole was closed with a 60 mm long linear staple with white cartilage in tangential direction. RY reconstruction was performed with an antecolic route and isoperistaltic Roux limb (length 30 cm) divided 20 cm from the ligament of Treitz. Side-to-side gastrojejunostomy and side-to-side jejunojejunostomy were performed intracorporeally with a 60 mm long linear stapler with white cartilage. The entry hole was closed by the same technique of Braun anastomosis. In the Roux-en-Y group, mesenteric defect was routinely repaired by a continuous suture with 3-0 Vicryl (Ethicon, Rome, Italy) or V-Loc 90 (Covidien, Mansfield, Massachusetts), whereas Petersen's defect was not repaired in both groups.

### 2.3. Statistical Analysis

All statistical analyses were performed using SPSS version 20.0 (SPSS Inc., Chicago, IL, USA). Differences between the two groups were assessed using *χ*^2^ tests, Fisher's exact tests, and Student's* t*-tests, as appropriate. A* p* value < 0.05 was considered statistically significant.

## 3. Results

Table 1 shows the demographic and clinical characteristics of the two groups. Age, sex, comorbidities, body mass index (BMI), American Society of Anesthesiologists (ASA) score, extent of surgery, number of retrieved lymph nodes, and pathologic stage were similar in the two groups.

Short-term surgical outcomes and postoperative complications are shown in Table 2. Operation time was significantly longer in the RY than in the BII Braun group (157.3 ± 33.9 min versus 134.6 ± 28.8 min,* p* < 0.010). Time to first sips of water (1.8 ± 0.5 versus 2.0 ± 0.9 days,* p* = 0.307) and length of hospital stay (7.9 ± 8.4 versus 7.0 ± 1.6 days,* p* = 0.583), however, did not differ between the two groups. There were also no significant differences in anesthesia time, blood loss, time to first flatus, and postoperative complications. Ileus occurred in three patients (10.0%) in the RY group.

**Figure 1:**
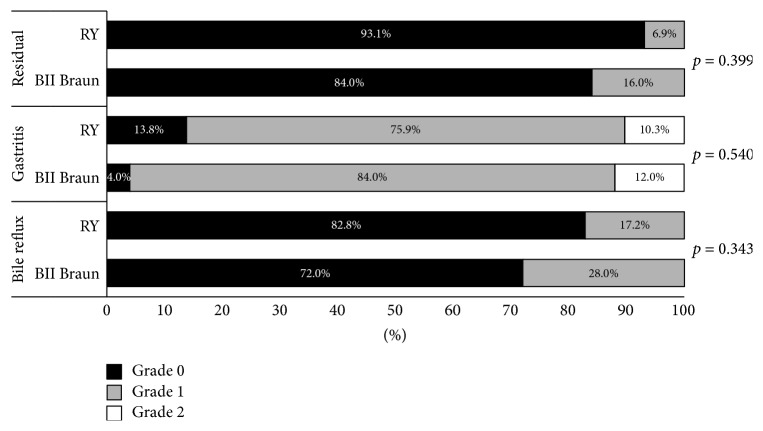
RGB score of gastroscopy at postoperative 6 months.

Functional outcomes were assessed indirectly by weight change and gastrointestinal symptoms (Table 3). The RY group showed a trend of larger weight loss than the BII Braun group, but body mass index of postoperative 3 and 6 months did not differ between the BII Braun and RY groups (22.2 versus 22.0,* p* = 0.842 and 21.9 versus 21.6,* p* = 0.680). Regarding GI symptoms, there was no significant difference in occurrence rate between two groups (11.5% in the BII Braun group versus 30.0% in the RY group, *p* = 0.114). Endoscopic finding was performed at 6 months after surgery in 25 patients (96.1%) in the BII Braun group and 29 (96.6%) in the RY group. The grades of gastric residue, remnant gastritis, and bile reflux did not differ in these two groups (Figure 1).

## 4. Discussion

Although the number of LDGs has increased worldwide since its introduction in the 1990s [23], there is a lack of consensus among surgeons regarding the choice of reconstructive procedure after LDG. The three methods, BI, BII, and RY, have advantages and disadvantages. The ideal gastrointestinal reconstruction procedure should minimize postoperative morbidity and improve quality of life.

A survey in Korea in 2009 found that BI was the most frequent type of reconstruction after distal gastrectomy (6581 patients, 63.4%), followed by BII reconstruction (3437 patients, 33.1%), with RY reconstruction rarely performed (332 patients, 3.3%) [24]. Increased experience with TLDG has increased the use of intracorporeal anastomosis, with KLASS 01 data showing that a significantly larger number of patients underwent BII reconstruction than RY following LDG (232 versus 20,* p* < 0.001) [25], because BII was both simpler and faster to perform. In Japan, however, the most common method of reconstruction was BI, followed by RY [26]. BII reconstruction was rarely performed by any Japanese surgeons, because it can cause more severe bile reflux, which may strongly correlate with carcinogenesis in the gastric remnant [26]. The results of our study suggest that BII Braun reconstruction after LDG for gastric cancer has perioperative outcomes similar to those of RY reconstruction, as shown by rates of postoperative complications and 6-month postoperative bile reflux (RGB) scores. These findings indicate that BII Braun anastomosis successfully diverts a substantial amount of bile from the remnant stomach and therefore may be an alternative to RY reconstruction in treating bile reflux.

Postoperative complications leading to malnutrition, such as delayed gastric emptying, anastomotic leak, and dumping syndrome, may require enteral nutritional support, prolong hospital stay, and increase health care costs. A comparison of patients who underwent BII or RY reconstruction found no differences in the rates of postgastrectomy diarrhea (9.1% versus 9.7%), dumping syndrome (6% versus 3.2%), and weight gain (78.8%* vs*. 90.3%) [27]. Although our retrospective registry did not include specific information on dumping syndrome and relevant quality-of-life parameters, we evaluated length of hospital stay, discharge destination, and hospital readmission as indirect measures of potential postoperative gastrointestinal dysfunction. We found all of these parameters were similar in the BII Braun and RY groups.

A retrospective study from Memorial Sloan Kettering Cancer Center compared outcomes in 122 patients who underwent RY reconstruction and 588 who underwent classic BII reconstruction after pancreaticoduodenectomy [28]. There were no differences in the rates of delayed gastric emptying (10.1% versus 10.3%), reoperation (9.1%* vs*. 6.9%), interventional radiology procedures (9.8% versus 6.8%), length of hospital stay (11 days versus 10 days), and mortality (0.9% versus 2.6%), findings similar to the results of our study.

Theoretically, LDG with BII Braun anastomosis may also minimize specific complications such as afferent loop syndrome and roux stasis syndrome. Braun anastomosis can divert a substantial amount of bile from the remnant stomach to the efferent loop; thereby it may reduce the afferent loop syndrome compared with BII without Braun anastomosis [29]. In our study, three patients in the RY group experienced ileus, indicating the roux stasis syndrome. Roux stasis syndrome is characterized by symptoms such as nausea, vomiting, epigastric pain, fullness, and difficulties in eating after Roux-en-Y gastrojejunostomy. However, the clinical definition of the roux stasis syndrome is ambiguous, sometimes confusing with postoperative ileus. Thus, more study is required to clarify the reality of roux stasis syndrome.

This study had several limitations, including its retrospective design. It was difficult to identify intraoperative factors that may have influenced the choice of BII Braun or RY reconstruction, but a certain selection bias might influence the present study. For instance, in the size of resected specimen, the mean length of greater curvature is significantly longer in the RY than in the BII Braun group (22.0 cm* vs.* 19.0 cm,* p* = 0.016). However, this was not a direct evidence that RY group has larger remnant stomach than BII Braun group and its clinical influence might be limited because the BMI were not different during postoperative 6 months. Moreover, the patient population was relatively small. Furthermore, it is difficult to discern retrospectively whether specific complications were directly related to the type of reconstruction. However, BII Braun anastomosis successfully diverted a substantial amount of bile from the remnant stomach, making this outcome comparable in the two groups.

## 5. Conclusions

The type of reconstruction after LDG had no effect on the rate or distribution of postoperative complications, length of hospital stay, or postoperative bile reflux scores. As BII Braun anastomosis successfully diverted a substantial amount of bile from the remnant stomach, this method may be a good alternative to RY reconstruction in preventing bile reflux. Short-term perioperative outcomes showed that BII Braun anastomosis and RY reconstruction can be considered equally acceptable restorative options following LDG for gastric cancer.

## Figures and Tables

**Table 1:** Clinicopathologic characteristics of the patients.

Variable	BII Braun (*n* = 26)	Roux-en-Y (*n* = 30)	*p* value
Age	60.1 ± 13.3	57.6 ± 12.6	0.466
Sex			0.218
Male	15 (57.7%)	22 (73.3%)	
Female	11 (42.3%)	8 (26.7%)	
BMI (kg/m^2^)	23.3 ± 3.3	24.0 ± 3.5	0.491
ASA score			0.453
1	13 (50.0%)	18 (60.0%)	
2	13 (50.0%)	12 (40.0%)	
Comorbidity	12 (46.2%)	12 (40.0%)	0.643
Hypertension	10	6	
Diabetes mellitus	6	7	
Liver diseases	3		
Tuberculosis	2	1	
Myocardial Infarction	1		
Extent of lymph node dissection			0.906
D1+	10 (38.5%)	12 (40.0%)	
D2	16 (61.5%)	18 (60.0%)	
Number of retrieved lymph nodes	36.7 ± 15.4	35.8 ± 10.9	0.793
Pathologic stage			0.043
I	12 (46.2%)	23 (76.6%)	
II	7 (26.9%)	5 (16.7%)	
III	7 (26.9%)	2 (6.7%)	

Values are presented as number (%) or mean ± standard deviation.

BMI = body mass index; ASA = American Society of Anesthesiologists.

**Table 2:** Comparison of surgical outcomes according to the reconstructive procedures.

Variable	BII Braun (*n* = 26)	Roux-en-Y (*n* = 30)	*p* value
Operating time (min)	134.6 ± 28.8	157.3 ± 33.9	0.010
Blood loss (ml)	89.2 ± 85.5	96.0 ± 89.8	0.773
Sips of water (d)	2.0 ± 0.9	1.8 ± 0.5	0.307
Soft diet (d)	3.2 ± 0.8	3.1 ± 0.8	0.784
Hospital stay (d)	7.0 ± 1.6	7.9 ± 8.4	0.583
Postoperative complications	4 (15.3%)	6 (20.0%)	0.653
Wound	1 (3.8%)		
Intraluminal bleeding	1 (3.8%)	1 (3.3%)	
Ileus		3 (10.0%)	
Leakage		1 (3.3%)	
Pancreatitis	1 (3.8%)	1 (3.3%)	
Other	1 (3.8%)		
*Clavien-Dindo* classification			1.000
Grades I-II	2 (7.6%)	3 (10.0%)	
Grades III-IV	2 (7.6%)	3 (10.0%)	

Values are presented as number (%) or mean ± standard deviation.

**Table 3:** Comparison of postoperative weight change and gastrointestinal symptoms.

Variable	BII Braun (*n* = 26)	Roux-en-Y (*n* = 30)	*p* value
Length of resected stomach (cm)			
Lesser curvature	12.4 ± 2.6	13.7 ± 2.6	0.086
Greater curvature	19.0 ± 3.9	22.0 ± 4.9	0.016
Body weight (kg)			
Preoperative	61.9 ± 10.7	67.3 ± 13.1	0.102
Postoperative 3 months	58.9 ± 10.3	61.8 ± 12.0	0.334
Postoperative 6 months	58.3 ± 10.3	60.7 ± 12.2	0.424
BMI (kg/m^2^)			
Preoperative	23.3 ± 3.3	24.0 ± 3.5	0.491
Postoperative 3 months	22.2 ± 3.1	22.0 ± 3.2	0.842
Postoperative 6 months	21.9 ± 3.0	21.6 ± 3.2	0.680
Postoperative GI symptoms	3 (11.5%)	9 (30.0%)	0.114
Diarrhea	3	3	
Constipation		2	
Dyspepsia		1	
Gas bloating		1	
Reflux/soreness		2	

Values are presented as number (%) or mean ± standard deviation.

BMI = body mass index; GI = gastrointestinal.
